# Psychedelic Renaissance: A New Paradigm in Mental Health

**DOI:** 10.1192/j.eurpsy.2026.11512

**Published:** 2026-07-31

**Authors:** A. M. Matas Ochoa, A. R. Quiroga, I. D. Sacristán

**Affiliations:** 1 Psiquiatría, Hospital Universitario Infanta Leonor; 2 Psiquiatría, Hospital Universitario Ramón y Cajal, Madrid, Spain

## Abstract

**Introduction:**

Psychedelics are hallucinogenic agents that are awakening scientific interest in recent years, especially in the field of mental health. Psychedelics induce non-ordinary states of consciousness and alterations in the emotions and in cognition and could be beneficial in treating some mental illness.

**Objectives:**

The objective of the poster is to expand knowledge about emerging treatments in mental health.

**Methods:**

Review of recent literature on psychedelics and their therapeutic uses. The studies were collected of the electronic databases PubMed.

**Results:**

Psychedelic-assisted psychotherapy (PAP) has emerged as an alternative strategy but also as an innovative drug class in treatment of depression, anxiety, posttraumatic stress disorder and other conditions. Different pharmacological agents have been studied, such as LSD, psilocybin, N,N-dimethyltryptamine (DMT), ayahuasca, or mescaline, but also 3,4-methylenedioxymethamphetamine (MDMA) and ketamine.

**Conclusions:**

Although preliminary findings, science support the improvement of some mental illnesses with psychedelics. Additionally, the minimal risk and low potential for abuse of these substances create a promising area of research.

**Disclosure of Interest:**

None Declared

